# Prediction of survival after neoadjuvant chemotherapy for breast cancer by evaluation of tumor-infiltrating lymphocytes and residual cancer burden

**DOI:** 10.1186/s12885-017-3927-8

**Published:** 2017-12-28

**Authors:** Yuka Asano, Shinichiro Kashiwagi, Wataru Goto, Koji Takada, Katsuyuki Takahashi, Takaharu Hatano, Satoru Noda, Tsutomu Takashima, Naoyoshi Onoda, Shuhei Tomita, Hisashi Motomura, Masahiko Ohsawa, Kosei Hirakawa, Masaichi Ohira

**Affiliations:** 1 Department of Surgical Oncology, Asahi-machi, Abeno-ku, Osaka, 545-8585 Japan; 2 Department of Pharmacology, Asahi-machi, Abeno-ku, Osaka, 545-8585 Japan; 3 Department of Plastic and Reconstructive Surgery, 1-4-3 Asahi-machi, Abeno-ku, Osaka, 545-8585 Japan; 4 Department of Diagnostic Pathology, Osaka City University Graduate School of Medicine, 1-4-3 Asahi-machi, Abeno-ku, Osaka, 545-8585 Japan

**Keywords:** Residual cancer burden, Tumor-infiltrating lymphocytes, Neoadjuvant chemotherapy, Breast cancer, Predictive marker

## Abstract

**Background:**

The tumor immune environment not only modulates the effects of immunotherapy, but also the effects of other anticancer drugs and treatment outcomes. These immune responses can be evaluated with tumor-infiltrating lymphocytes (TILs), which has frequently been verified clinically. On the other hand, residual cancer burden (RCB) evaluation has been shown to be a useful predictor of survival after neoadjuvant chemotherapy (NAC). In this study, RCB and TILs evaluations were combined to produce an indicator that we have termed “RCB-TILs”, and its clinical application to NAC for breast cancer was verified by subtype-stratified analysis.

**Methods:**

A total of 177 patients with breast cancer were treated with NAC. The correlation between RCB and TILs evaluated according to the standard method, and prognosis, including the efficacy of NAC, was investigated retrospectively. The RCB and TILs evaluations were combined to create the “RCB-TILs”. Patients who were RCB-positive and had high TILs were considered RCB-TILs-positive, and all other combinations were RCB-TILs-negative.

**Results:**

On multivariable analysis, being RCB-TILs-positive was an independent factor for recurrence after NAC in all patients (*p* < 0.001, hazard ratio = 0.048), triple-negative breast cancer (TNBC) patients (*p* = 0.018, hazard ratio = 0.041), HER2-positive breast cancer (HER2BC) patients (*p* = 0.036, hazard ratio = 0.134), and hormone receptor-positive breast cancer (HRBC) patients (*p* = 0.002, hazard ratio = 0.081).

**Conclusions:**

The results of the present study suggest that RCB-TILs is a significant predictor for breast cancer recurrence after NAC and may be a more sensitive indicator than TILs alone.

**Electronic supplementary material:**

The online version of this article (doi: 10.1186/s12885-017-3927-8) contains supplementary material, which is available to authorized users.

## Background

Treatment with neoadjuvant chemotherapy (NAC) increases the rate of breast-conserving surgery and reduces the risk of postoperative recurrence in patients with resectable breast cancer [1–4]. The main purposes of NAC are to facilitate tumor regression, improve breast conservation rates, evaluate therapeutic effects, and establish therapeutic strategies based on the evaluation results [1, 5, 6]. Recently, NAC has required tailoring, particularly by exploring biomarkers using genetic approaches or establishing therapeutic strategies based on the response to early treatment. Although previous studies have described the prediction of survival after NAC by means of the pathological complete response (pCR) rate, tumor-infiltrating lymphocytes (TILs), and residual cancer burden (RCB), none of these have yet come into use in actual clinical practice [7–12].

Cancer cells have various gene abnormalities that allow them to proliferate spontaneously and survive, but the surrounding environment (cancer microenvironment) also influences cancer cells and is involved in the intrinsic characteristics of cancer [13]. The tumor immune environment not only influences the effects of immunotherapy but also the effects of other anticancer drugs and treatment outcomes [1, 14]. Thus, the importance of inhibiting and improving the tumor immune microenvironment is now recognized. TILs are regarded as an indicator for monitoring such immune responses, and studies have found that they are prognostic factors and predictors of response to treatment in a range of types of cancer [15, 16]. A large amount of evidence has now been reported for the clinical relevance of the morphological evaluation of TILs in breast cancer, and the subject is now attracting attention [9, 15–18]. We have previously reported the clinical validity and utility of the evaluation of TILs in NAC [19].

RCB evaluation has been shown to be a useful predictor of survival after NAC [11, 12]. RCB after NAC is calculated by a method developed by Symmans and colleagues at the University of Texas MD Anderson Cancer Center [11]. One study that used this calculation method for the analysis of survival after NAC found that, for the triple-negative breast cancer (TNBC) and hormone receptor-positive breast cancer (HRBC) subtypes, RCB evaluation was useful for predicting long-term survival [12].

TILs are also believed to be useful markers for predicting response to treatment in the TNBC and human epidermal growth factor receptor-2 (HER2)-positive breast cancer (HER2BC) subtypes, which are associated with high levels of immune activity [20]. We therefore hypothesized that combining the evaluation of TILs with that of RCB might provide a sensitive indicator that is also capable of predicting survival in HRBC. In this study, RCB and TILs evaluations were combined to produce an indicator that we have termed “RCB-TILs”, and its clinical application to NAC for breast cancer was verified by subtype-stratified analysis.

## Methods

### Patient background

This study was conducted at Osaka City University Graduate School of Medicine, Osaka, Japan, according to the Reporting Recommendations for Tumor Marker prognostic Studies (REMARK) guidelines and a retrospectively written research, pathological evaluation, and statistical plan. Written, informed consent was obtained from all patients. This research conformed to the provisions of the Declaration of Helsinki of 2013. The study protocol was approved by the Ethics Committee of Osaka City University (#926).

A total of 177 patients with resectable, early-stage breast cancer diagnosed as stage IIA (T1, N1, M0 or T2, N0, M0), IIB (T2, N1, M0 or T3, N0, M0), or IIIA (T1–2, N2, M0 or T3, N1–2, M0) were treated with NAC between 2007 and 2013. Tumor stage and T and N factors were stratified based on the TNM Classification of Malignant Tumors, UICC Seventh Edition [21]. Our previous reports have also used the same patient population and the present study, but it was the study of the significance of CD8 /FOXP3 ratio or androgen receptor [19, 22]. Breast cancer was confirmed histologically by core needle biopsy and staged by systemic imaging studies using computed tomography (CT), ultrasonography (US), and bone scintigraphy. Breast cancer was classified into subtypes according to the immunohistochemical expressions of estrogen receptor (ER), progesterone receptor (PgR), HER2, and Ki67. Based on their immunohistochemical expression profiles, tumors are categorized into immunophenotypes: luminal A (ER+ and/or PgR+, HER2-, Ki67-low); luminal B (ER+ and/or PgR+, HER2+) (ER+ and/or PgR+, HER2-, Ki67-high), HER2-enriched (HER2BC) (ER-, PgR-, and HER2+); and TNBC (negative for ER, PgR, and HER2) [23]. In this study, luminal A and luminal B were considered hormone receptor-positive breast cancer (HRBC).

All patients received a standardized protocol of NAC consisting of four courses of FEC100 (500 mg/m^2^ fluorouracil, 100 mg/m^2^ epirubicin, and 500 mg/m^2^ cyclophosphamide) every 3 weeks, followed by 12 courses of 80 mg/m^2^ paclitaxel administered weekly [24, 25]. Forty-five patients had HER2-positive breast cancer and were given additional weekly (2 mg/kg) or tri-weekly (6 mg/kg) trastuzumab during paclitaxel treatment [26]. All patients underwent chemotherapy as outpatients. Therapeutic anti-tumor effects were assessed according to the Response Evaluation Criteria in Solid Tumors (RECIST) criteria [27]. Patients underwent mastectomy or breast-conserving surgery after NAC. The pathological effect of chemotherapy was assessed for resected primary tumors after NAC. Pathological complete response (pCR) was defined as the complete disappearance of the invasive components of the lesion with or without intraductal components, including in the lymph nodes, according to the National Surgical Adjuvant Breast and Bowel Project (NSABP) B-18 protocol [1]. All patients who underwent breast-conserving surgery underwent postoperative radiotherapy to the remnant breast. The standard postoperative adjuvant therapy for the subtype concerned was administered.

Overall survival (OS) time was the period from the initiation of NAC to the time of death from any cause. Disease-free survival (DFS) was defined as freedom from all local, loco-regional, and distant recurrences. All patients were followed-up by physical examination every 3 months, US every 6 months, and CT and bone scintigraphy annually. The median follow-up period was 3.4 years (range, 0.6–6.0 years) for the assessment of OS and 3.1 years (range, 0.1–6.0 years) for DFS. The primary end point of this study was DFS, and the secondary endpoint was OS and pCR rate.

### Histopathological evaluation of TILs

Histopathological assessment of predictive factors was performed on core needle biopsy (CNB) specimens at the time of the breast cancer diagnosis. In this study, TILs were evaluated in the same method as our previous studies [28]. Histopathological parameters examined included nuclear grade, histological type, presence of TILs, and correlations of these parameters with intrinsic subtypes and pCR.

Histopathologic analysis of the percentage of TILs was evaluated on a single full-face hematoxylin and eosin (HE)-stained tumor section using criteria described by Salgado et al. [29]. TILs were defined as the infiltrating lymphocytes within tumor stroma and were expressed by the proportion of the field investigated, and the number of TILs in stroma surrounding the stained cancer cells was quantitatively measured in each field under 400-times magnification [30, 31]. The areas of in situ carcinoma and crush artifacts were not included. Proportional scores of 3, 2, 1, and 0 were given if the area of stroma containing lymphoplasmacytic infiltration around invasive tumor cell nests comprised >50%, >10–50%, ≤10%, and 0%, respectively. A score of ≥2 was considered positive for TILs, whereas scores of 1 and 0 were considered negative. Histopathologic evaluation of TILs was jointly performed by two breast pathologists, who were blinded to clinical information, including treatment allocation and outcomes.

### Histopathological evaluation of RCB

The RCB was calculated using the Residual Cancer Burden Calculator on the website of the MD Anderson Cancer Center [11]. This automatically calculates the RCB on the basis of data on the primary tumor (primary tumor bed area, overall cancer cellularity, and percentage of cancer that is in situ disease) and lymph node metastasis (number of positive lymph nodes and diameter of largest metastasis). The RCB is categorized into one of three classes: minimal residual disease (RCB-I), moderate residual disease (RCB-II), or extensive residual disease (RCB-III). Since RCB-I is considered to have a better prognosis than RCB-II and RCB-III, RCB-I was considered positive, and RBC-II and RCB-III were considered negative.

### RCB-TILs evaluation

The RCB and TILs evaluations were combined to create the “RCB-TILs”. Patients who were RCB-I-positive and had positive TILs were considered RCB-TILs-positive, and all other combinations were RCB-TILs-negative.

### Statistical analysis

Statistical analysis was performed using the SPSS version 19.0 statistical software package (IBM, Armonk, NY, USA). The associations between TILs, RCB-TILs, and clinicopathological variables were examined using χ^2^ tests. Multivariable analysis of pCR was carried out using a binary logistic regression model. The Kaplan-Meier method was used to estimate DFS and OS, and the results were compared between groups with log-rank tests. A Cox proportional hazards model was used to compute univariable and multivariable hazards ratios (HR) for the study parameters with 95% confidence intervals (c.i.), and a backward stepwise method was used for variable selection in multivariable analyses. A *p* value <0.05 was considered significant. Cutoff values for different biomarkers included in this study were chosen before statistical analysis.

## Results

### RCB-TILs and clinicopathological investigation

Of the patients who underwent NAC, 112 (63.3%) were RCB-TILs-positive, and 65 (36.7%) were negative. RCB-TILs-positive patients had a significantly higher nuclear grade (*p* = 0.034), higher Ki67 value (*p* = 0.001), higher proportion of TNBC (*p* = 0.001), lower proportion of HRBC (*p* < 0.001), and a higher pCR rate (*p* < 0.001) (Table 1). A further investigation within each subtype was performed. Among the 61 patients with TNBC, RCB-TILs-positive patients had a significantly higher pCR rate (*p* = 0.023), whereas among HER2BC patients, RCB-TILs-positive patients had a significantly lower pCR rate (*p* = 0.004). In HRBC patients, RCB-TILs-positive patients had a significantly higher nuclear grade (*p* = 0.004), higher Ki67 value (*p* = 0.024), and higher pCR rate (*p* = 0.007) (Table 2).

**Table 1:** Correlation between clinicopathological features and RCB-TILs in 177 breast cancers

Parameters	RCB-TILs in all breast cancers (*n* = 177)		p value
	Positive (*n* = 112)	Negative (*n* = 65)	
Age at operation			
≤ 56	52 (46.4%)	35 (53.9%)	
> 56	60 (53.6%)	30 (46.1%)	0.341
Menopause			
Pre-menopausal	44 (39.3%)	28 (43.1%)	
Post-menopausal	68 (60.7%)	37 (56.9%)	0.621
Tumor size			
≤ 2 cm	19 (17.0%)	5 (7.7%)	
> 2 cm	93 (83.0%)	60 (92.3%)	0.082
Lymph node status			
Negative	27 (24.1%)	14 (21.5%)	
Positive	85 (75.9%)	51 (78.5%)	0.696
Nuclear grade			
1, 2	81 (72.3%)	56 (86.2%)	
3	31 (27.7%)	9 (13.8%)	0.034
Ki67			
≤ 14%	36 (32.1%)	38 (58.5%)	
> 14%	76 (67.9%)	27 (41.5%)	0.001
Intrinsic subtype			
TNBC	49 (43.8%)	12 (16.0%)	
non-TNBC	63 (56.2%)	53 (84.0%)	0.001
Intrinsic subtype			
HER2BC	26 (23.2%)	10 (15.4%)	
non- HER2BC	86 (76.8%)	55 (84.6%)	0.212
Intrinsic subtype			
HRBC	37 (33.0%)	43 (66.2%)	
non-HRBC	75 (67.0%)	22 (33.8%)	<0.001
Pathological response			
pCR	58 (51.8%)	9 (13.8%)	
non-pCR	54 (48.2%)	56 (86.2%)	<0.001

*RCB* residual cancer burden, *TILs* tumor-infiltrating lymphocytes, *TNBC* triple-negative breast cancer, *HER2BC* human epidermal growth factor receptor 2-enriched breast cancer, *HRBC* hormone receptor-positive breast cancer, *pCR* pathological complete response

**Table 2:** Correlations between RCB-TILs and clinicopathological parameters in 61 triple-negative, 36 HER2-positive, and 80 hormone receptor-positive breast cancers

Parameters	TNBC (*n* = 61)		*p* value	HER2BC (*n* = 36)		*p* value	HRBC (*n* = 80)		*p* value
	Positive (*n* = 49)	Negative (*n* = 12)		Positive (*n* = 26)	Negative (*n* = 10)		Positive (*n* = 37)	Negative (*n* = 43)	
Age at operation									
≤ 56	23 (46.9%)	5 (41.7%)		12 (46.2%)	4 (40.0%)		17 (45.9%)	26 (60.5%)	
> 56	26 (53.1%)	7 (58.3%)	0.743	14 (53.8%)	6 (60.0%)	0.519	20 (50.1%)	17 (39.5%)	0.194
Menopause									
Pre-menopausal	17 (34.7%)	5 (41.7%)		11 (42.3%)	3 (30.0%)		16 (43.2%)	20 (46.5%)	
Post-menopausal	32 (65.3%)	7 (58.3%)	0.652	15 (57.7%)	7 (70.0%)	0.389	21 (56.8%)	23 (53.5%)	0.770
Tumor size									
≤ 2 cm	7 (14.3%)	0 (0.0%)		5 (19.2%)	1 (10.0%)		7 (18.9%)	4 (9.3%)	
> 2 cm	42 (85.7%)	12 (100.0%)	0.197	21 (80.8%)	9 (90.0%)	0.456	30 (81.1%)	39 (90.7%)	0.179
Lymph node status									
Negative	9 (18.4%)	2 (16.7%)		8 (30.8%)	3 (30.0%)		10 (27.0%)	9 (20.9%)	
Positive	40 (81.6%)	10 (83.3%)	0.630	18 (69.2%)	7 (70.0%)	0.647	27 (73.0%)	34 (79.1%)	0.353
Nuclear grade									
1, 2	37 (75.5%)	7 (58.3%)		19 (73.1%)	9 (90.0%)		25 (67.6%)	40 (93.0%)	
3	12 (24.5%)	5 (41.7%)	0.234	7 (26.9%)	1 (10.0%)	0.269	12 (32.4%)	3 (7.0%)	0.004
Ki67									
≤ 14%	13 (26.5%)	5 (41.7%)		10 (38.5%)	7 (70.0%)		13 (35.1%)	26 (60.5%)	
> 14%	36 (73.5%)	7 (58.3%)	0.303	16 (61.5%)	3 (30.0%)	0.090	24 (64.9%)	17 (39.5%)	0.024
Pathological response									
pCR	26 (53.1%)	2 (16.7%)		9 (34.6%)	9 (90.0%)		15 (40.5%)	6 (14.0%)	0.007
non-pCR	23 (46.9%)	10 (83.3%)	0.023	17 (65.4%)	1 (10.0%)	0.004	22 (59.5%)	37 (86.0%)	

*RCB* residual cancer burden, *TILs* tumor-infiltrating lymphocytes, *TNBC* triple-negative breast cancer, *HER2BC* human epidermal growth factor receptor 2-enriched breast cancer, *HRBC* hormone receptor-positive breast cancer, *pCR* pathological complete response

### Analysis of survival according to RCB-TILs

**Fig. 1:**
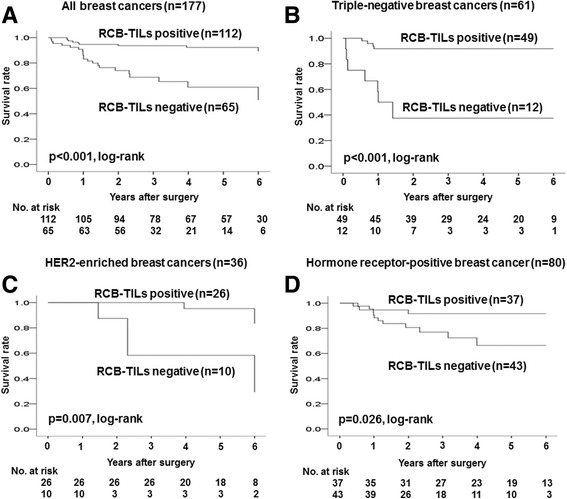
Analysis of RCB-TILs status and outcome in breast cancer (Disease Free Survival, DFS). Survival was analyzed according to RCB-TILs. DFS after NAC was significantly longer for RCB-TILs-positive patients than for RCB-TILs-negative patients in all patients (*p* < 0.001, log-rank) (**a**), TNBC patients (*p* < 0.001, log-rank) (**b**), HER2BC patients (*p* = 0.007, log-rank) (**c**), and HRBC patients (*p* = 0.026, log-rank) (**d**)

Survival was analyzed according to RCB-TILs. DFS after NAC was significantly longer for RCB-TILs-positive patients than for RCB-TILs-negative patients in all patients (*p* < 0.001, log-rank), TNBC patients (*p* < 0.001, log-rank), HER2BC patients (*p* = 0.007, log-rank), and HRBC patients (*p* = 0.026, log-rank) (Fig. 1a-d). Overall survival was significantly longer for RCB-TILs-positive patients than for RCB-TILs-negative patients in all patients (*p* = 0.005, log-rank) and TNBC patients (*p* < 0.001, log-rank), but the difference was not significant for HER2BC patients (*p* = 0.585, log-rank) or HRBC patients (*p* = 0.128, log-rank) (Additional file 1: Figure S1A–D).

**Fig. 2:**
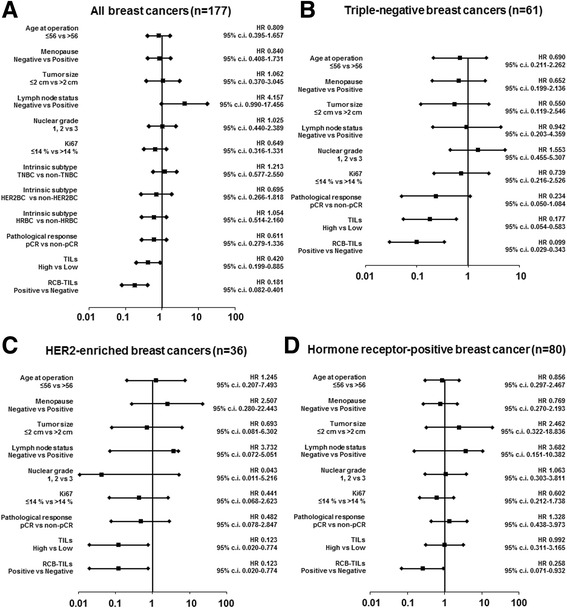
Forest plots. Univariable analysis of patients with being RCB-TILs-positive found that this contributed significantly to prolonging DFS in all patients (*p* < 0.001, hazard ratio = 0.181) (**a**), TNBC patients (*p* < 0.001, hazard ratio = 0.099) (**b**), HER2BC patients (*p* = 0.026, hazard ratio = 0.123) (**c**), and HRBC patients (*p* = 0.039, hazard ratio = 0.258) (**d**)

Univariable analysis of patients with high TILs found that this contributed significantly to prolonging DFS in all patients (*p* = 0.022, HR = 0.420), TNBC patients (*p* = 0.004, HR = 0.177), and HER2BC patients (*p* = 0.026, HR = 0.123). For HRBC patients, however, high TILs did not contribute to survival (*p* = 0.990, HR = 0.992). Being RCB-TILs-positive, however, contributed significantly to prolonging DFS in all patients (*p* < 0.001, HR = 0.181), TNBC patients (*p* < 0.001, HR = 0.099), HER2BC patients (p = 0.026, HR = 0.123), and HRBC patients (*p* = 0.039, HR = 0.258) (Table 3, Fig. 2a-d).

**Table 3:** Univariable and multivariable analysis with respect to disease-free survival in breast cancer subtypes

		Univariable analysis			Multivariable analysis		
Parameter		Hazard ratio	95% c.i.	*p* value	Hazard ratio	95% c.i.	*p* value
All breast cancers (*n* = 177)							
Age	≤56 vs >56	0.809	0.395–1.657	0.561			
Menopause	Pre- vs Post-	0.840	0.408–1.731	0.637			
Tumor size (cm)	≤2 vs >2	1.062	0.370–3.045	0.911			
Lymph node status	Negative vs Positive	4.157	0.990–17.456	0.052			
Nuclear grade	1–2 vs 3	1.025	0.440–2.389	0.954			
Ki67 (%)	≤14 vs >14	0.649	0.316–1.331	0.238			
Intrinsic subtype	TNBC vs non-TNBC	1.213	0.577–2.550	0.611			
Intrinsic subtype	HER2BC vs non- HER2BC	0.695	0.266–1.818	0.459			
Intrinsic subtype	HRBC vs non-HRBC	1.054	0.514–2.160	0.886			
Pathological response	pCR vs non-pCR	0.611	0.279–1.336	0.217	1.008	0.402–2.524	0.987
TILs	High vs Low	0.420	0.199–0.885	0.022	4.785	1.169–19.582	0.029
RCB-TILs	Positive vs Negative	0.181	0.082–0.401	<0.001	0.048	0.012–0.188	<0.001
TNBC (*n* = 61)							
Age	≤56 vs >56	0.690	0.211–2.262	0.541			
Menopause	Pre- vs Post-	0.652	0.199–2.136	0.480			
Tumor size (cm)	≤2 vs >2	0.550	0.119–2.546	0.444			
Lymph node status	Negative vs Positive	0.942	0.203–4.359	0.939			
Nuclear grade	1–2 vs 3	1.553	0.455–5.307	0.482			
Ki67 (%)	≤14 vs >14	0.739	0.216–2.526	0.630			
Pathological response	pCR vs non-pCR	0.234	0.050–1.084	0.063	0.270	0.030–2.466	0.246
TILs	High vs Low	0.177	0.054–0.583	0.004	0.243	0.071–0.816	0.023
RCB-TILs	Positive vs Negative	0.099	0.029–0.343	<0.001	0.041	0.003–0.573	0.018
HER2BC (*n* = 36)							
Age	≤56 vs >56	1.245	0.207–7.493	0.811			
Menopause	Pre- vs Post-	2.507	0.280–22.443	0.411			
Tumor size (cm)	≤2 vs >2	0.693	0.081–6.302	0.744			
Lymph node status	Negative vs Positive	3.732	0.072–5.051	0.414			
Nuclear grade	1–2 vs 3	0.043	0.011–5.216	0.513			
Ki67 (%)	≤14 vs >14	0.441	0.068–2.623	0.364			
Pathological response	pCR vs non-pCR	0.482	0.078–2.847	0.415	0.702	0.108–4.551	0.710
TILs	High vs Low	0.123	0.020–0.774	0.026	0.134	0.020–0.879	0.036
RCB-TILs	Positive vs Negative	0.123	0.020–0.774	0.026	0.134	0.020–0.879	0.036
HRBC (*n* = 80)							
Age	≤56 vs >56	0.856	0.297–2.467	0.773			
Menopause	Pre- vs Post-	0.769	0.270–2.193	0.623			
Tumor size (cm)	≤2 vs >2	2.462	0.322–18.836	0.386			
Lymph node status	Negative vs Positive	3.682	0.151–10.382	0.205			
Nuclear grade	1–2 vs 3	1.063	0.303–3.811	0.930			
Ki67 (%)	≤14 vs >14	0.602	0.212–1.738	0.344			
Pathological response	pCR vs non-pCR	1.328	0.438–3.973	0.614	2.123	0.667–6.750	0.202
TILs	High vs Low	0.992	0.311–3.165	0.990	1.044	0.323–3.372	0.949
RCB-TILs	Positive vs Negative	0.258	0.071–0.932	0.039	0.081	0.016–0.409	0.002

*c.i* confidence interval, *TILs* tumor-infiltrating lymphocytes, *RCB* residual cancer burden, *TNBC* triple-negative breast cancer, *HER2BC* human epidermal growth factor receptor 2-enriched breast cancer, *HRBC* hormone receptor-positive breast cancer, *pCR* pathological complete response

**Fig. 3:**
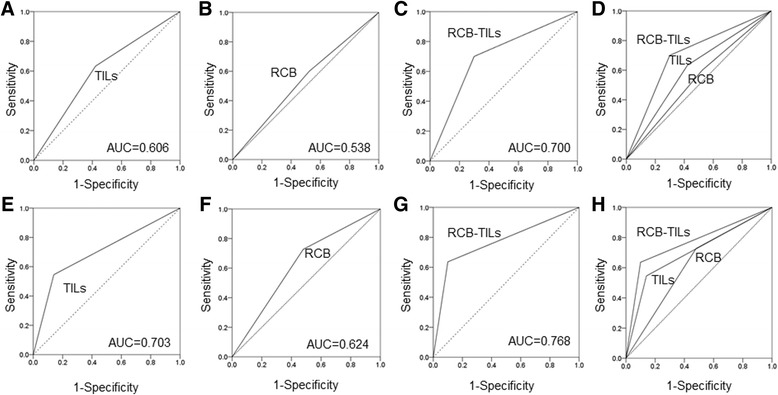
On ROC curve analyses in all breast cancer and TNBC patients. ROC analysis showed that, for all breast cancer patients, the results for the RCB-TILs (AUC: 0.700) were better than those for the TILs (AUC: 0.606) and the RCB (AUC: 0.538) (**a**–**d**). ROC analysis for TNBC patients also found similar results (AUC: TILs = 0.703, RCB = 0.624, RCB-TILs = 0.768) (**e**-**h**)

**Fig. 4:**
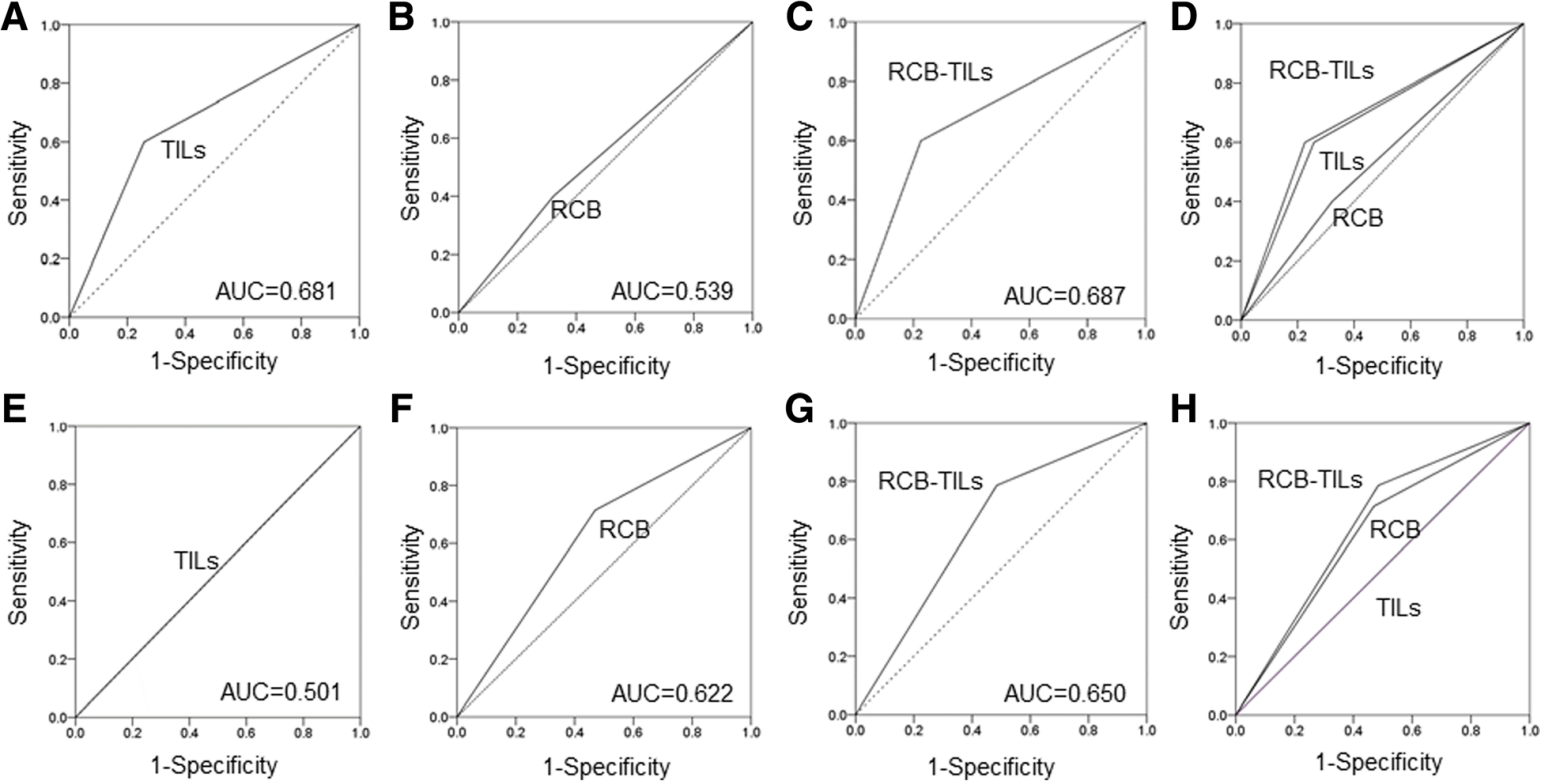
On ROC curve analyses in HER2BC and HRBC patients. ROC analysis showed that, for HER2BC patients, the results for the RCB-TILs (AUC: 0.687) were better than those for the TILs (AUC: 0.681) and the RCB (AUC: 0.539) (**a**–**d**). ROC analysis for HRBC patients also found similar results (AUC: TILs = 0.501, RCB = 0.622, RCB-TILs = 0.650) (**e**-**h**)

Receiver operating characteristic (ROC) analysis showed that, for all breast cancer patients, the results for the RCB-TILs [area under the curve (AUC): 0.700] were better than those for the TILs (AUC: 0.606) and RCB (AUC: 0.538) (Fig. 3a–d). An analysis by subtype also found similar results for TNBC patients (AUC: TILs = 0.703, RCB = 0.624, RCB-TILs = 0.768) (Fig. 3e-h), HER2BC patients (AUC: TILs = 0.681, RCB = 0.539, RCB-TILs = 0.687) (Fig. 4a–d), and HRBC patients (AUC: TILs = 0.501, RCB = 0.622, RCB-TILs = 0.650) (Fig. 4e–h).

On multivariable analysis, high TILs was an independent factor contributing to prolonging DFS in all patients (*p* = 0.029, HR = 4.785), TNBC patients (*p* = 0.023, HR = 0.243), and HER2BC patients (*p* = 0.036, HR = 0.134). For HRBC patients, however, no contribution to survival (*p* = 0.949, HR = 1.044) was observed. Being RCB-TILs-positive was an independent factor for recurrence after NAC in all patients (*p* < 0.001, HR = 0.048), TNBC patients (*p* = 0.018, HR = 0.041), HER2BC patients (*p* = 0.036, HR = 0.134), and HRBC patients (*p* = 0.002, HR = 0.081) (Table 3).

## Discussion

The definition of pCR after NAC is based on tumor infiltration or non-infiltration and the status of the axillary lymph nodes [32]. DFS is clearly improved for patients who have achieved pCR as a result of NAC compared with non-pCR patients, and this is considered to be of major significance [32, 33]. However, although pCR does contribute to survival in highly malignant breast cancers such as TNBC and HER2BC, it has been shown that it does not provide an indicator of survival in the low-malignancy subtype of HRBC [32, 34]. In the prediction of response to treatment, TILs evaluation is also only predictive of response to treatment with NAC in TNBC and HER2BC patients [9, 16, 18]. The subtype for which it is the most difficult to predict the response to treatment with NAC is thus HRBC, which is the most common. RCB evaluation after NAC, on the other hand, has been found to be useful for predicting survival in HRBC patients [11, 12]. RCB-TILs, our proposed indicator, was useful for predicting survival to post-NAC recurrence in all subtypes.

TILs is regarded as a marker of subtypes with high immune activity, while pCR is considered to be a marker of subtypes with high cellular proliferation activity [7–9, 35]. In HRBC patients, RCB-TILs-positive patients had a significantly higher Ki67 value and higher pCR rate. In this study, the RCB-TILs-positive HRBC cases were found to have high immune activity and high cellular proliferation activity. When we combined the markers useful for the various different subtypes to create a new method of evaluation in terms of RCB-TILs, we were able to predict survival after NAC for patients with all of the various subtypes. We also showed that this is a more sensitive indicator than prediction by TILs alone. In the choice of additional treatment after NAC, RCB-TILs evaluation may thus contribute to treatment strategies that are neither excessive nor inadequate. However, this study had the limitations of being a retrospective investigation and of differences in adjuvant therapy after NAC. Clinical trials of CREAT-X and other adjuvant therapies after NAC are currently being reported [36]. It is to be hoped that such clinical trials will also investigate the validity of RCB-TILs for predicting survival after NAC.

There are some subtypes of HRBC for which endocrine therapy is relatively ineffective. In this study, all HRBC patients were treated with postoperative endocrine therapy. However, RCB-TILs-negative patients had a high rate of recurrence, suggesting that RCB-TILs may provide a marker for predicting the response to endocrine therapy. A new treatment strategy is conceivable whereby RCB-TILs-positive HRBC patients undergo conventional endocrine therapy after NAC while additional chemotherapy is chosen for those who are RCB-TILs-negative.

## Conclusions

The results of the present study suggest that RCB-TILs is a significant predictor for breast cancer recurrence after NAC and may be a more sensitive indicator than TILs alone.

## Additional file

Additional file 1: Figure S1. Analysis of RCB-TILs status and outcome in breast cancer (Overall Survival, OS). OS was significantly longer for RCB-TILs-positive patients than for RCB-TILs-negative patients in all patients (*p* = 0.005, log-rank) (**A**) and TNBC patients (*p* < 0.001, log-rank) (**B**), but the difference was not significant for HER2BC patients (*p* = 0.585, log-rank) (**C**) or HRBC patients (*p* = 0.128, log-rank) (**D**). (ZIP 154 kb)

## Abbreviations

AUC: Area under the curve
c.i: Confidence interval
CNB: Core needle biopsy
CT: Computed tomography
DFS: Disease-free survival
ER: Estrogen receptor
HE: Hematoxylin and eosin
HER: Human epidermal growth factor receptor
HER2BC: HER2-enriched
HR: Hazard ratio
HRBC: Hormone receptor-positive breast cancer
NAC: Eoadjuvant chemotherapy
NSABP: National surgical adjuvant breast and bowel project
OS: Overall survival
pCR: Pathological complete response
PgR: Progesterone receptor
RCB: Residual cancer burden
RECIST: Response evaluation criteria in solid tumors
REMARK: Reporting recommendations for tumor marker prognostic studies
ROC: Receiver operating characteristic
TILs: Tumor-infiltrating lymphocytes
TNBC: Triple-negative breast cancer
TS: Training Set
UICC: Union for international cancer control
US: Ultrasonography
VS: Validation Set
