# Supplementary figures and images for: Prediction of survival after neoadjuvant chemotherapy for breast cancer by evaluation of tumor-infiltrating lymphocytes and residual cancer burden
Source: BMC Cancer. 2017 Dec 28;17:888. doi: 10.1186/s12885-017-3927-8 (PMC5745786; doi:10.1186/s12885-017-3927-8)

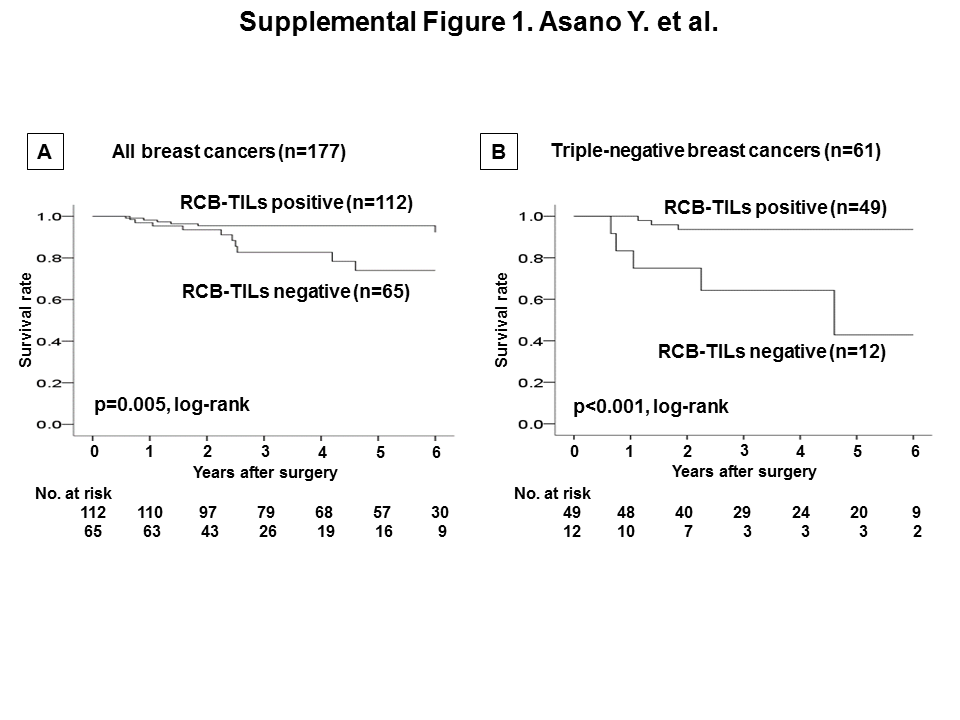

Supplement: Additional file 1: Figure S1. — Analysis of RCB-TILs status and outcome in breast cancer (Overall Survival, OS). OS was significantly longer for RCB-TILs-positive patients than for RCB-TILs-negative patients in all patients (p = 0.005, log-rank) (A) and TNBC patients (p < 0.001, log-rank) (B), but the difference was not significant for HER2BC patients (p = 0.585, log-rank) (C) or HRBC patients (p = 0.128, log-rank) (D). (ZIP 154 kb) [file 12885_2017_3927_MOESM1_ESM.zip › Suppl Fig.1A-BR3.TIF]

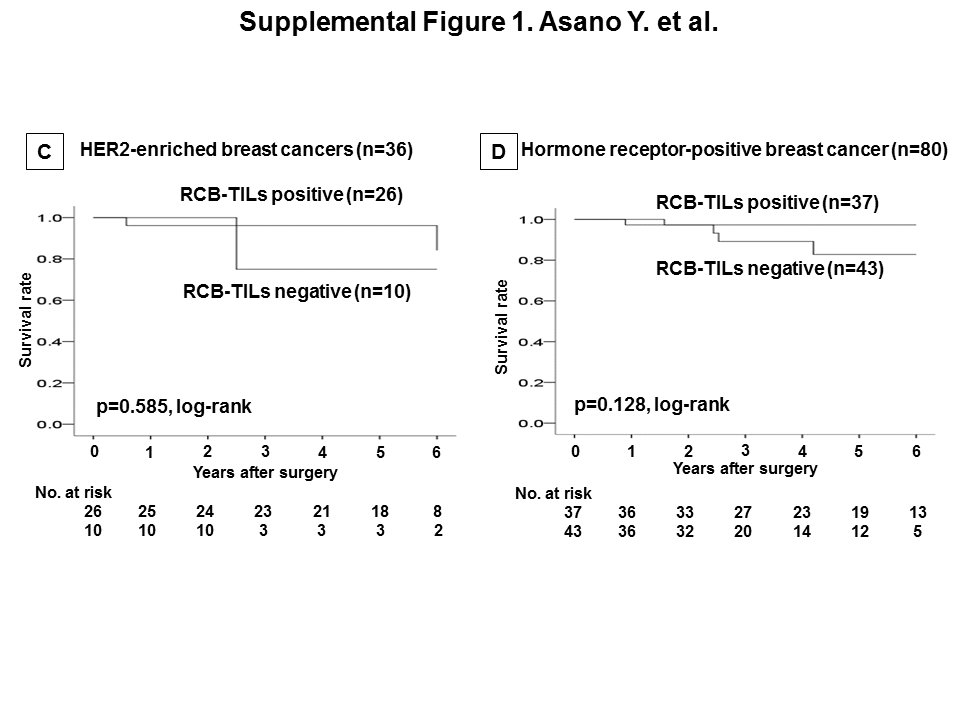

Supplement: Additional file 1: Figure S1. — Analysis of RCB-TILs status and outcome in breast cancer (Overall Survival, OS). OS was significantly longer for RCB-TILs-positive patients than for RCB-TILs-negative patients in all patients (p = 0.005, log-rank) (A) and TNBC patients (p < 0.001, log-rank) (B), but the difference was not significant for HER2BC patients (p = 0.585, log-rank) (C) or HRBC patients (p = 0.128, log-rank) (D). (ZIP 154 kb) [file 12885_2017_3927_MOESM1_ESM.zip › Suppl Fig.1C-DR3.TIF]
